# Population genetics and forensic efficiency of 30 InDel markers in four Chinese ethnic groups residing in Sichuan

**DOI:** 10.1080/20961790.2020.1737470

**Published:** 2020-04-21

**Authors:** Fei Wang, Guanglin He, Zheng Wang, Mengge Wang, Jing Liu, Xing Zou, Shouyu Wang, Mengyuan Song, Ziwei Ye, Mingkun Xie, Yiping Hou

**Affiliations:** Institute of Forensic Medicine, West China School of Basic Medical Sciences & Forensic Medicine, Sichuan University, Chengdu, China

**Keywords:** Forensic sciences, InDel, population genetics, Sichuan Han, Sichuan Yi, Sichuan Tibetan, Sichuan Hui

## Abstract

Sichuan Province is located at the transitional junction regions of the Qinghai-Tibet Plateau and the low-altitude plains. It also serves as the corridor of Sino-Tibetan-speaking population migration and expansion since neolithic expansion of Proto-Tibeto-Burman populations from Middle/Upper Yellow River during Majiayao period (3300–2000 BC). However, the population structure and the corresponding genetic diversity of forensic-related markers in this region remain unclear. Thus, we genotyped 30 insertion-deletion (InDel) markers in 444 samples from four ethnic groups (Han, Tibetan, Hui and Yi) from Sichuan Province using the Investigator^®^ DIPplex kit to explore the characteristics of population genetics and forensic genetic focuses. All the loci were found to be in Hardy-Weinberg Equilibrium (HWE) after applying a Bonferroni correction and no pairwise loci showed prominent linkage disequilibrium. The combined matching probability (CMP) and the combined power of discrimination (CPD) are larger than 1.8089 × 10^−11^ and 0.99999999995, respectively. Principal component analysis, multi-dimensional scaling plots and Neighbour-Joining tree among 65 worldwide populations indicated that Sichuan Hui and Han are genetically close to Hmong-Mien and Tai-Kadai-speaking populations, and Sichuan Tibetan and Yi bear a strong genetic affinity with Tibeto-Burman-speaking populations. The model-based genetic structure further supports the genetic affinity between the studied populations and linguistically close populations.

**Key Points**
- Forensic parameters of 30 insertion-deletions (InDels) in 444 individuals from four populations are reported, which showed abundant genetic affinity and diversity among populations and high value in personal identification.
- Genetic similarities existed between the studied populations and ethnically, linguistically close populations.
- Sichuan Hui and Han are genetically close to Hmong-Mien and Tai-Kadai-speaking populations.
- Sichuan Tibetan and Yi bear a strong genetic affinity with Tibeto-Burman-speaking populations.

## Introduction

Insertion-deletion (InDel) polymorphisms has been a promising and powerful supplementary tool in forensic personal identification cases and kinship testing, since they bear the advantages of lower mutation rate, smaller amplicons and absence of stutter peaks compared with traditional gold standard short tandem repeats (STRs) [1–5].

Lying in Southwest China, backland of the Eurasian continent, Sichuan Province owns a complex topography spanning Qinghai-Tibet Plateau, Hengduan Mountains, Yunnan-Guizhou Plateau, Qinba Mountains and Sichuan Basin. Evidence of both archaeology and linguistics subsequently suggested that these regions play an important role in the formation of modern Sino-Tibetan-speaking populations, especially for Tibeto-Burman-speaking populations spreading southwards and westwards from the middle and upper basins of Yellow River during Yangshao period (about 7 000–5 000 years BP) with the millet cultivation expansion [6, 7].

Nowadays, the permanent population of Sichuan has exceeded 80 000 000 according to Sichuan Population Statistics Bulletin 2018 (http://tjj.sc.gov.cn/tjxx/zxfb/201903/t20190319_277119.html), which is nearly equivalent to Germany’s population. As a multi-ethnic province, Sichuan is the biggest agglomeration for the Yi ethnic group, the second-largest community of Tibetan nationality and residence for large number of Hui group. However, Han group is still the largest population here (93.53%). Over the past hundreds of years, the ethnic groups have been the largest immigrant population inflow and accounted for an indispensable part in the construction of genetic variability in the province. In this study, we use the Investigator^®^ DIPplex kit (Qiagen, Hilden, Germany) to investigate population genetics and forensic efficiency in these four Sichuan ethnic groups.

## Materials and methods

### Sample collection

Bloodstains were collected from 444 healthy unrelated individuals from Sichuan Province (155 Chengdu Hans, 132 Liangshan Yis, 119 Tibetans of Ganzi and Aba, and 38 Chengdu Huis) after obtained participants’ written informed consents with the approval of the Ethics Committee of the Institute of Forensic Medicine, Sichuan University (K2015008).

### DNA extraction, quantification, amplification and genotyping

Genomic DNA was extracted with the DNA Blood Mini Kit (Qiagen) and quantified using the NanoDrop-2000 Spectrophotometers (Thermo Fisher Scientific, Waltham, MA, USA) according to the manufacturer’s instructions. InDels amplification was carried out using the Investigator^®^ DIPplex PCR Amplification Kit (Qiagen) following the manufacturer’s protocols and analyzed with Applied Biosystems 3130 Genetic Analyzer and Gene Mapper v3.2 (Thermo Fisher).

### Quality control

Control DNA 9948 (Qiagen) and ddH_2_O (Qiagen) were amplified and genotyped along the population samples as the positive and negative control, respectively. All experiments were carried out in our laboratory which has been accredited by the China National Accreditation Service for Conformity Assessment (CNAS) and ISO 17025.

### Reference database

To comprehensively explore the patterns of genetic affinity between our studied populations and worldwide reference populations, we merged our newly obtained population data with those of 61 worldwide populations. The final database used in the population comparison study included 6483 East Asian individuals from 38 populations [4, 5, 8–26], 494 South Asians from three populations [27–29], 300 Vietnamese of Southeast Asia [30], 194 West Asians from two populations [31], 621 African individuals from five populations [27, 30, 32], 984 European individuals from eight populations [4, 31–35] and 1 071 South Americans from eight populations [36, 38]. The detailed geographical regions and population sizes of these included populations are submitted in Supplementary Table S1.

### Analysis of data

Allele frequencies and forensic efficiency parameters were calculated using online software STRAF (http://cmpg.unibe.ch/shiny/STRAF/) [39]. The heat map was carried out by R Statistical Software v3.0.2 (https://www.r-project.org/). Multi-dimensional scaling analysis (MDS) was performed by SPSS software v25.0 (IBM Corporation, Armonk, NY, USA). Principal component analysis (PCA) was conducted using the MultiVariate Statistical Package (MVSP, Provalis Research, Montreal, Quebec, Canada). Neighbour-joining tree (N-J tree) was carried out by the Molecular Evolutionary Genetics Analysis (MEGA, https://www.megasoftware.net/) on basis of the Nei’s standard genetic distances (R_st_) using the Phylogeny Inference Package (PHYLIP, http://evolution.genetics.washington.edu/phylip.html). The genetic structure composition analysis was performed by the STRUCTURE programme v2.3.4 (https://web.stanford.edu/group/pritchardlab/structure.html) [40].

## Results and discussion

### Forensic characteristics

All InDel markers were successfully amplified and genotyped in the 444 samples with no significant deviation from Hardy-Weinberg Equilibrium (HWE) and no significant linkage disequilibrium after performing a Bonferroni correction (*P* ≤ 0.0017) (see raw genotype data in Supplementary Table S2). Allele frequencies, forensic parameters, and efficiencies in the four Sichuan populations are shown in Supplementary Table S3. The highest power of discrimination (PD) loci were HLD88 (PD = 0.6519) in Sichuan Han, HLD40 (PD = 0.6294) in Sichuan Yi, HLD124 (PD = 0.6405) in Sichuan Tibetan and HLD88 (PD = 0.6607) in Sichuan Hui. The combined matching probability (CMP) and the combined power of discrimination (CPD) ranged from 1.8089 × 10^−11^ to 4.6696 × 10^−11^, 0.99999999995330 to 0.99999999998191, respectively. The highest and lowest combined powers of paternity exclusion (CPE) were 0.9919 in Sichuan Hui and 0.9861 in Sichuan Tibetan. These observed values indicated that the InDel system can serve as a powerful supplementary tool for existing STR system.

### Population genetic analyses

To further explore the genetic similarities and differences between populations, 61 worldwide population data of 30 InDel markers were collected into the reference database mentioned above [4, 5, 8–38]. The heat map was built based on the insertion allele frequencies (DIP+) of 30 InDels from four Sichuan populations and 61 previously published populations, which was shown in Supplementary Figure S1. On the left side of the heat map, 65 populations were separated into two clusters: Mexican populations and others. The second cluster was divided into two branches as African populations (Somali, Nigerian, Xhosa, Zulu) and others. The further separation happened between Asian, European and American roughly. In most Asian populations, which is contrasted with other populations, HLD111, HLD39 and HLD122 loci show prominent low insertion frequencies and HLD118, HLD99, HLD64 and HLD81 loci perform distinct high frequencies.

**Figure 1.**
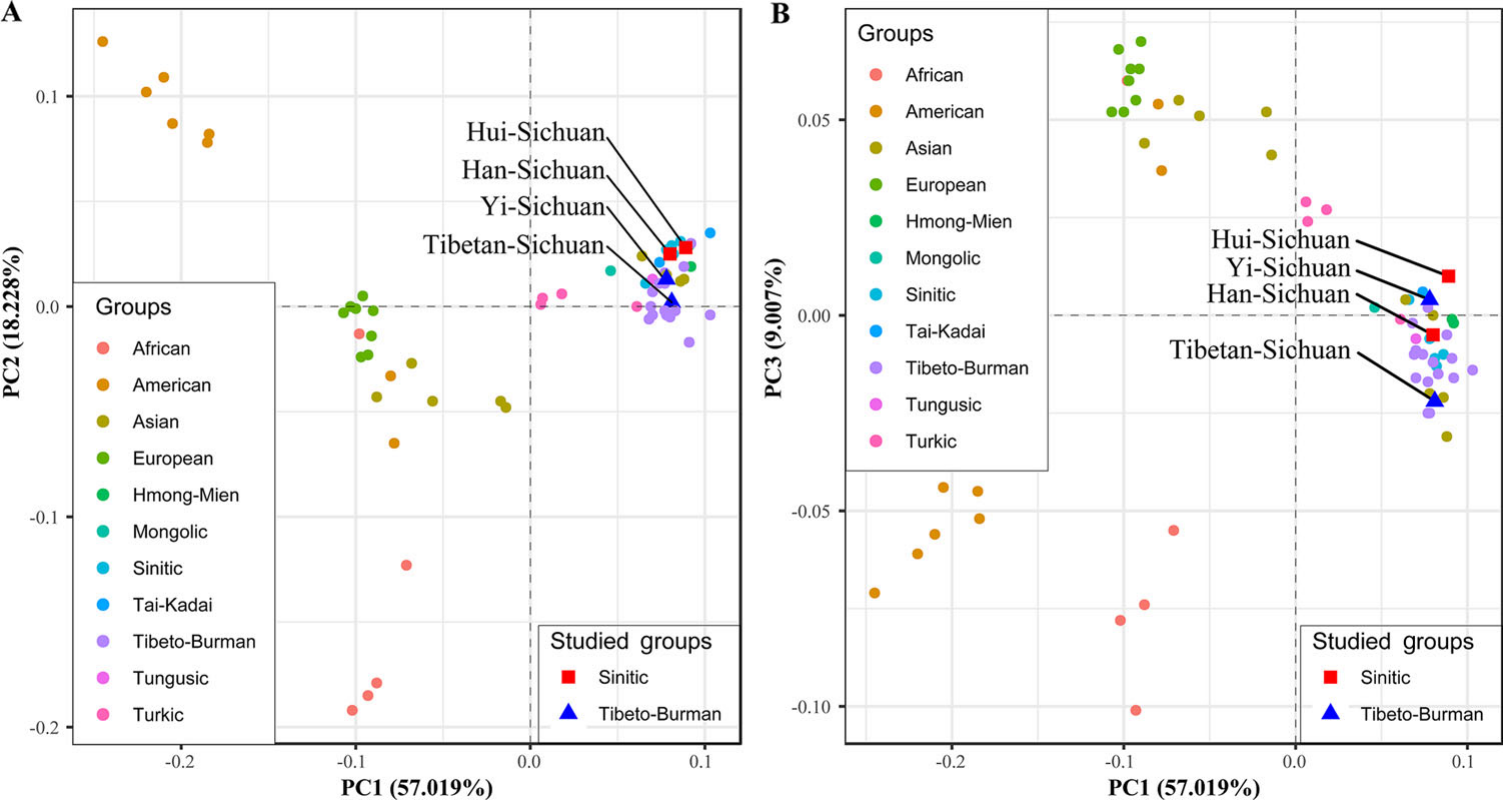
Principal components analysis among 65 worldwide populations revealed by top three components (PC1 = 57.019%, PC2 = 18.228%, PC3 = 9.007%), which explains 84.254% of the total differences.

MDS, PCA and N-J tree were generated based on the R_st_ generated from PHYLIP v.3.6. (Supplementary Table S4). As is shown in MDS (Supplementary Figure S2), population stratification along the continental geographical boundaries is identifiable. Genetic similarities and differences were further dissected using PCA (Figure 1). Top three components could explain 84.254% of the total differences (PC1 = 57.019%, PC2 = 18.228%, PC3 = 9.007%). Yi and Tibetan from Sichuan are genetically close to Tibeto-Burman-speaking populations (Yunnan Yi, Tibetan from Tibet, Qinghai and Sichuan, Tujia from Hubei), while Hui and Han are localized close with Hmong-Mien and Tai-Kadai-speaking populations (Zhuang, Dong and Miao from Guangxi).

The evolutionary genetic relationship revealed by the N-J tree (Supplementary Figure S3) follows a pattern of ethnic, geographical and linguistic affinity. Asian populations are close to each other on the large scale, while the Sichuan Tibetan group remains close to other 10 Tibetan groups, and Sichuan Hui group stays next to Xinjiang Hui in the picture. Guangdong Han is considerably closer to the geographically close Tai-Kadai or Hmong-Mien-speaking populations (Dong, Miao, and Zhuang from Guangxi).

**Figure 2.**
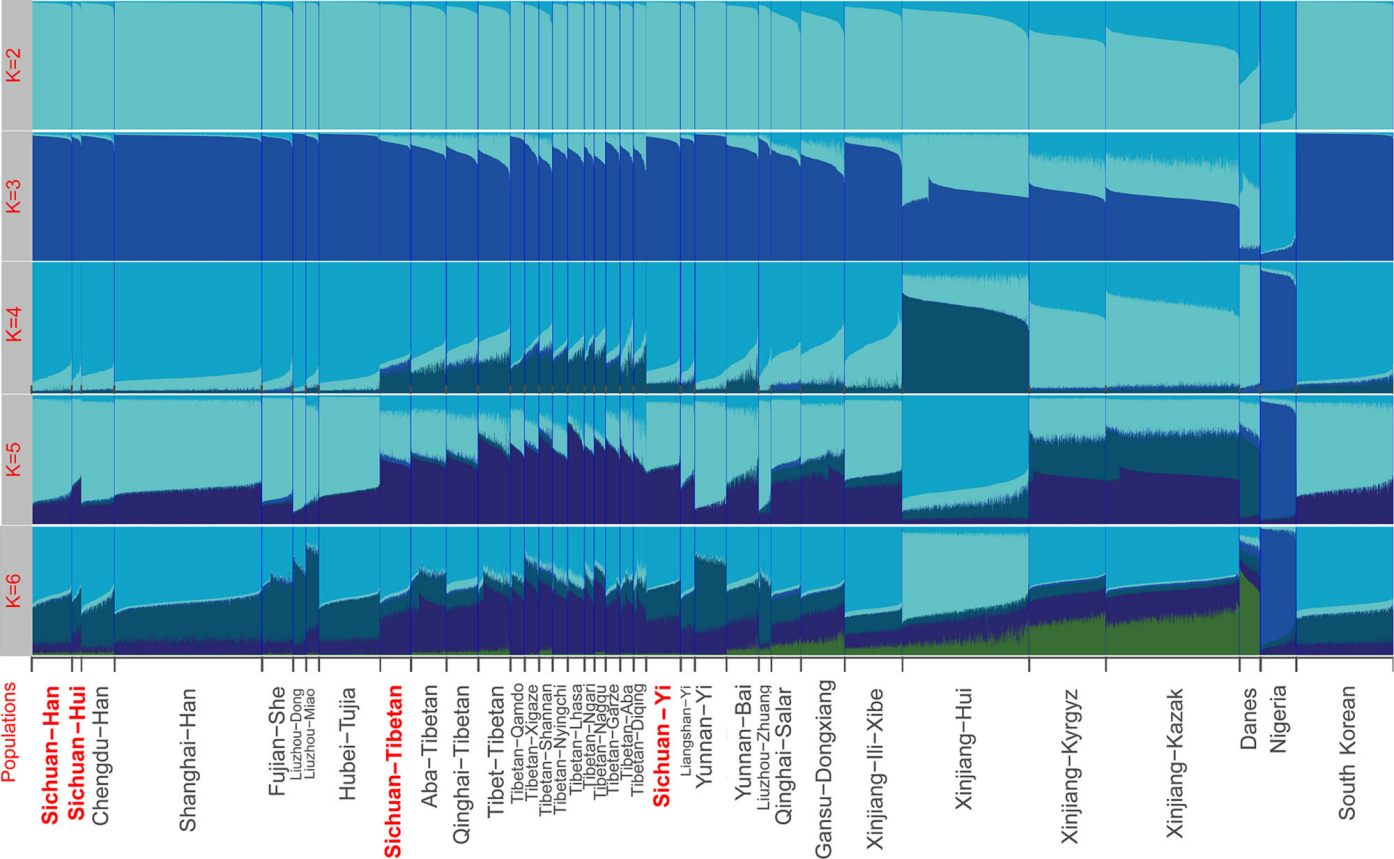
STRUCTURE analysis results of 36 worldwide populations on basis of their raw genotypes when the number of predefined ancestry (K) varies from 2 to 6 and the best K is 4.

The STRUCTURE analysis was used to dissect the genetic structure of the four Sichuan populations we studied and other 32 populations on the basis of their raw genotypes, which were shown in Figure 2 [8, 10, 12–17, 19, 21–26, 30, 33]. The K represents the number of the predefined ancestry. Genetic affinity in linguistically close populations can be identified when K increases from 5 to 6. Four investigated populations share large number of ancestry components with neighbouring East Asian populations, which was in accordance with results of MDS, PCA and N-J tree. Furthermore, as we all know, the Silk Road, the earliest and most important channel of communication between eastern and western civilizations, also served as a precious opportunity for gene communication between the populations along the road. Xinjiang was the midpoint of the road. As a result, Turkish-speaking groups (Xinjiang Hui, Kazak, and Kyrgyz) bear both Asian and European ancestries and appear as the interim of genetic structure between populations from East Asia and Europe when K equals 6.

## Conclusion

According to the obtained results, Han, Yi, Tibetan, and Hui group of Sichuan were scattered among the Asian groups in terms of genetic distance, evolutionary relationship and genetic structure. The least significant differences in the genetic relationship were found between studied populations and ethnically, linguistically close populations. Furthermore, based on the acquired values, the Investigator DIPplex^®^ PCR Amplification Kit has been proved to be extremely useful as a supplement of STR to apply in forensic case works in the four populations we studied.

## Supplementary Material

Supplemental Material
