# Supplementary figures and images for: Population genetics and forensic efficiency of 30 InDel markers in four Chinese ethnic groups residing in Sichuan
Source: Forensic Sci Res. 2020 Apr 21;7(3):498–502. doi: 10.1080/20961790.2020.1737470 (PMC9639512; doi:10.1080/20961790.2020.1737470)

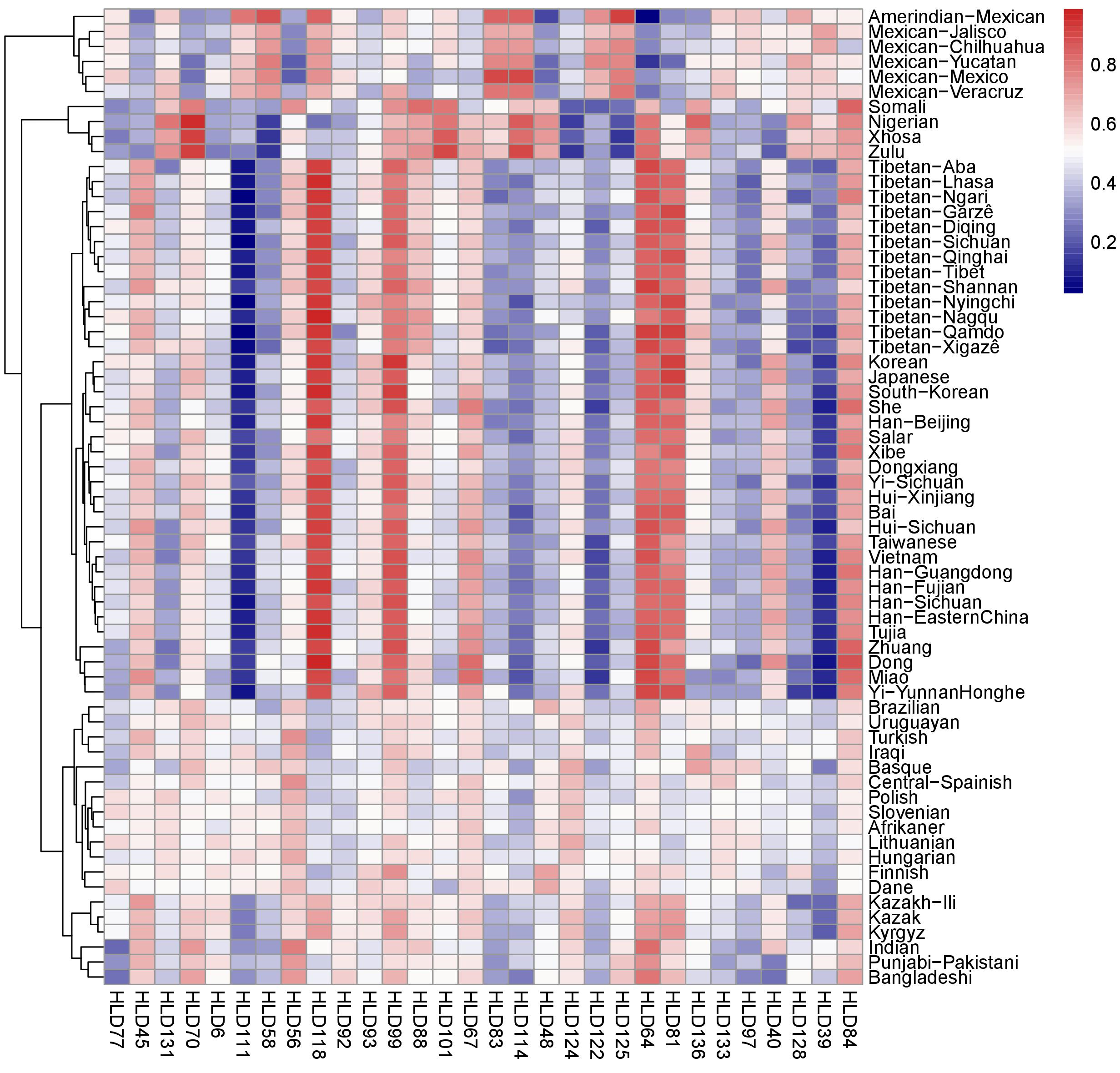

Supplement: Supplemental Material [file TFSR_A_1737470_SM5482.zip › TFSR_A_1737470_Supplementary_material/Supplementary Figure S1.tif]

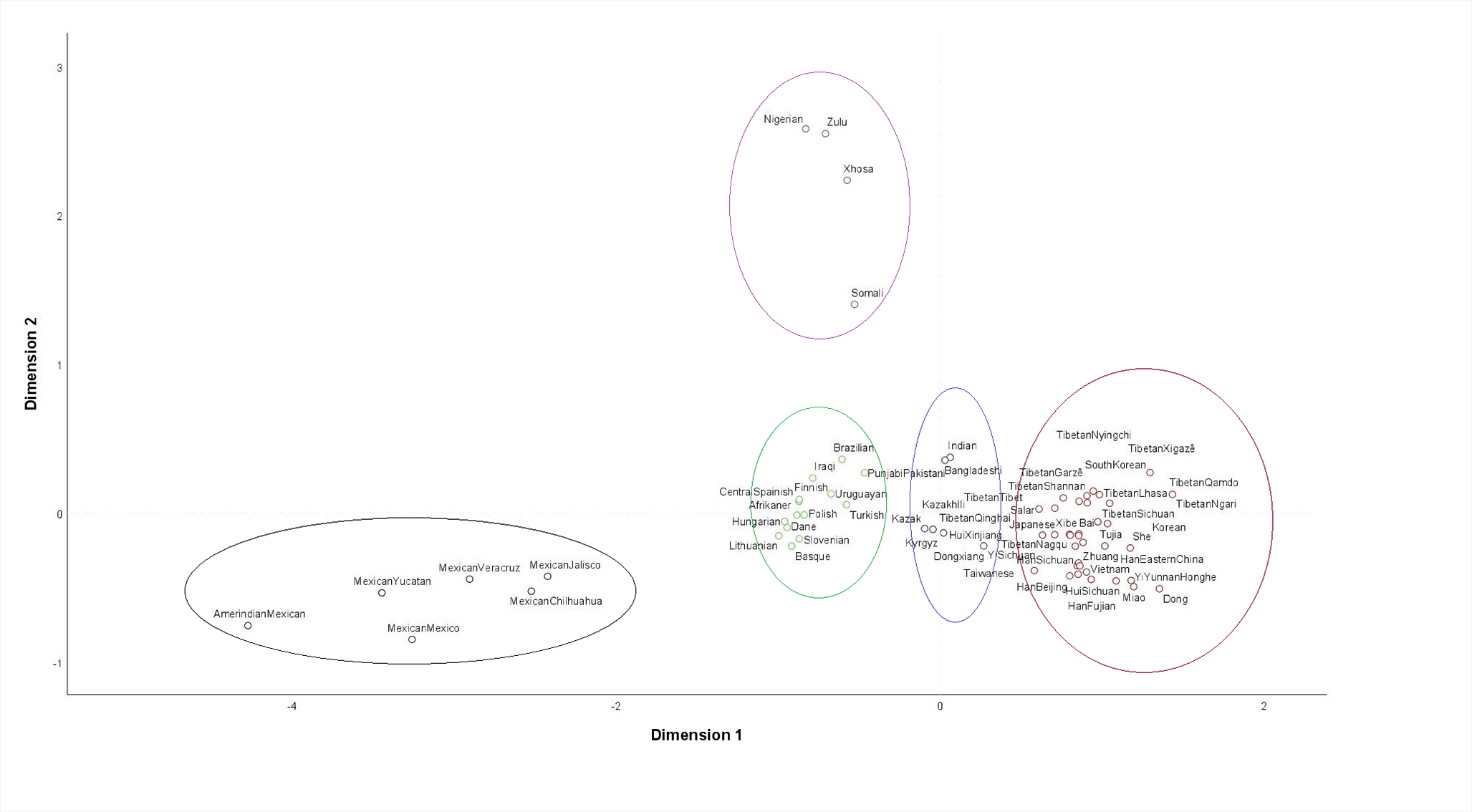

Supplement: Supplemental Material [file TFSR_A_1737470_SM5482.zip › TFSR_A_1737470_Supplementary_material/Supplementary Figure S2.tif]

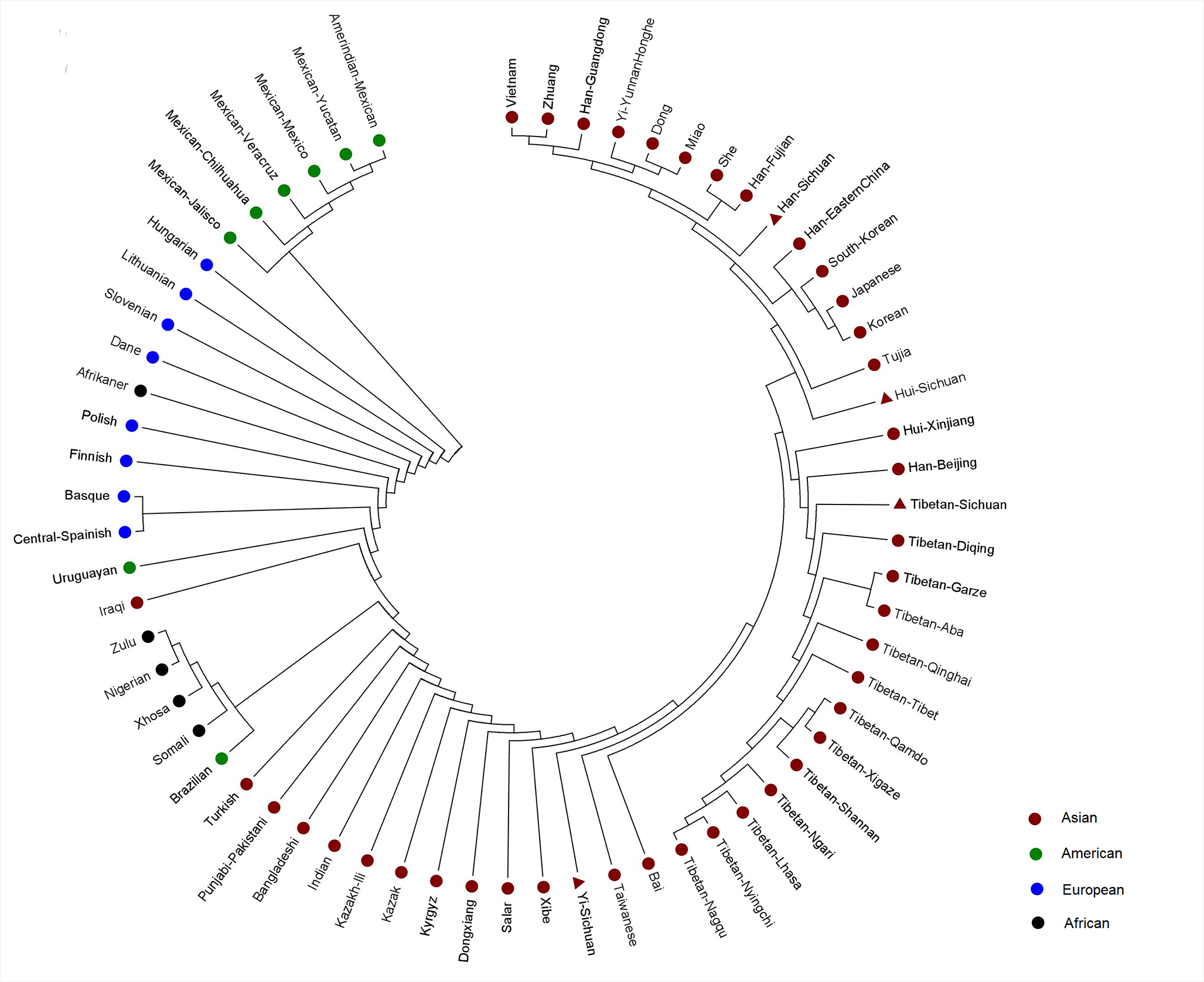

Supplement: Supplemental Material [file TFSR_A_1737470_SM5482.zip › TFSR_A_1737470_Supplementary_material/Supplementary Figure S3.tif]
